# TRPV4 functional status in cystic cells regulates cystogenesis in autosomal recessive polycystic kidney disease during variations in dietary potassium

**DOI:** 10.14814/phy2.15641

**Published:** 2023-03-22

**Authors:** Kyrylo Pyrshev, Anna Stavniichuk, Viktor N. Tomilin, Naghmeh Hassanzadeh Khayyat, Guohui Ren, Mariya Kordysh, Oleg Zaika, Mykola Mamenko, Oleh Pochynyuk

**Affiliations:** ^1^ Department of Integrative Biology and Pharmacology The University of Texas Health Science Center at Houston Houston Texas USA; ^2^ Department of Physiology Augusta University Augusta Georgia USA

**Keywords:** [Ca^2+^]_*i*_ signaling, cAMP, mechanosensitivity, PCK453 rats, renal injury

## Abstract

Mechanosensitive TRPV4 channel plays a dominant role in maintaining [Ca^2+^]_*i*_ homeostasis and flow‐sensitive [Ca^2+^]_*i*_ signaling in the renal tubule. Polycystic kidney disease (PKD) manifests as progressive cyst growth due to cAMP‐dependent fluid secretion along with deficient mechanosensitivity and impaired TRPV4 activity. Here, we tested how regulation of renal TRPV4 function by dietary K^+^ intake modulates the rate of cystogenesis and mechanosensitive [Ca^2+^]_*i*_ signaling in cystic cells of PCK453 rats, a homologous model of human autosomal recessive PKD (ARPKD). One month treatment with both high KCl (5% K^+^) and KB/C (5% K^+^ with bicarbonate/citrate) diets significantly increased TRPV4 levels when compared to control (0.9% K^+^). High KCl diet caused an increased TRPV4‐dependent Ca^2+^ influx, and partial restoration of mechanosensitivity in freshly isolated monolayers of cystic cells. Unexpectedly, high KB/C diet induced an opposite effect by reducing TRPV4 activity and worsening [Ca^2+^]_*i*_ homeostasis. Importantly, high KCl diet decreased cAMP, whereas high KB/C diet further increased cAMP levels in cystic cells (assessed as AQP2 distribution). At the systemic level, high KCl diet fed PCK453 rats had significantly lower kidney‐to‐bodyweight ratio and reduced cystic area. These beneficial effects were negated by a concomitant administration of an orally active TRPV4 antagonist, GSK2193874, resulting in greater kidney weight, accelerated cystogenesis, and augmented renal injury. High KB/C diet also exacerbated renal manifestations of ARPKD, consistent with deficient TRPV4 activity in cystic cells. Overall, we demonstrate that TRPV4 channel activity negatively regulates cAMP levels in cystic cells thus attenuating (high activity) or accelerating (low activity) ARPKD progression.

## Graphical abstract

Polycystic kidney disease (PKD) is a devastating pathology, which is characterized by the development of numerous fluid‐filled cysts leading to declined renal function. Previous evidence found augmented cAMP levels and impaired mechanosensitivity in cystic cells. Here, we investigate how functional activity of the mechanosensitive TRPV4 channel affects cystogenesis and cAMP levels in PCK453 rats, a homologous model of human autosomal recessive PKD. We show that high KCl diet increases renal TRPV4 levels, augments TRPV4‐dependent Ca^2+^ influx in freshly isolated cystic cell monolayers and markedly slow the development and growth of renal cysts in PCK453 rats. In contrast, impaired TRPV4 activity in cystic cells leads to the membrane accumulation of AQP2 water channel indicative of augmented cAMP levels in cystic cells leading to greatly accelerated cystogenesis and renal injury. We propose that pharmacological or dietary means leading to TRPV4 stimulation might be useful to counteract PKD progression in the clinical setting.

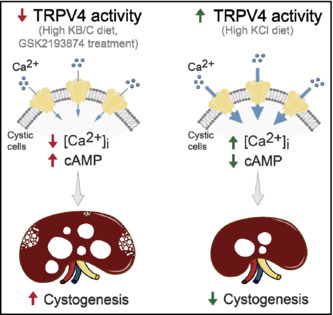

## 1 INTRODUCTION

Whole body water and solute homeostasis depends critically on the ability of the kidneys to perpetually filter plasma and to excrete any unneeded extras and waste products with urine. This is achieved by reabsorption of approximately 99% of the filtrate by the renal tubules accompanied by the secretion of certain constituents, most commonly potassium and protons. It is now generally accepted that dynamic alterations in fluid flow serve as physiologically relevant mechanical cues leading to elevations in intracellular Ca^2+^ concentration ([Ca^2+^]_*i*_) in renal tubule cells (Weinbaum et al., 2010). This, in turn, triggers numerous intracellular cascades to adjust the rates of reabsorption and secretion (Satlin et al., 2006; Weinbaum et al., 2010; Woda et al., 2001). The mechanosensitive Ca^2+^‐permeable transient receptor potential vanilloid type 4 (TRPV4) channel is abundantly expressed in the distal segments of the renal tubule, including the connecting tubule and the collecting duct, and also to a lesser extent in the proximal tubule (Berrout et al., 2012; Pyrshev et al., 2022). TRPV4 activation by increases in tubular flow/increased perfusion is indispensable for the direct Ca^2+^ influx and mechanosensitive elevations in [Ca^2+^]_*i*_ (Pyrshev et al., 2022). Genetic deletion of TRPV4 does not only abolish flow‐dependent [Ca^2+^]_*i*_ responses (Berrout et al., 2012), but also precludes flow‐induced alterations in electrolyte transport in perfused cortical collecting ducts (Taniguchi et al., 2007).

The term “polycystic kidney disease” entails a large diverse group of hereditary disorders, which commonly manifest as the formation and further enlargement of multiple fluid‐filled cysts in renal parenchyma (Bergmann et al., 2018, Ghata & Cowley, 2017). PKD progression causes a gradual decline of renal function and the development of interstitial fibrosis eventually progressing to the end‐stage renal disease (ESRD; Bergmann et al., 2018; Ghata & Cowley, 2017). Despite the distinct underlying genetic defects: polycystin 1 and 2 (PC1 and PC2) for the most prevalent autosomal dominant PKD (ADPKD) and fibrocystin for a rapidly evolving “juvenile” autosomal recessive PKD (ARPKD), the malicious epithelial‐to‐cyst cell transformation is associated with a drastic switch in overall transport direction from predominantly reabsorptive to secretory (Belibi et al., 2004; Sullivan et al., 1998). Abundant published evidence identified the central role of inappropriately increased intracellular cAMP levels leading to over‐activation of B‐Raf/ERK signaling in driving apical Cl^−^‐secretion and accelerated proliferation/apoptosis of cystic cells (Belibi et al., 2004; Wallace, 2011; Yamaguchi et al., 2003, 2006). Indeed, the only FDA‐approved drug for ADPKD treatment, vasopressin receptor 2 blocker, tolvaptan, interferes with cystogenesis by reducing intracellular cAMP levels although at the expense of compromised urinary concentrating ability (Edwards et al., 2018; Torres et al., 2004; van Gastel & Torres, 2017). This encourages further search of new strategies to decrease cAMP levels with no disturbance in systemic water balance during PKD treatment.

Multiple reports demonstrate that dysregulated cAMP signaling is associated with decreased basal [Ca^2+^]_*i*_ levels and inability to respond to flow‐induced shear stress by elevating [Ca^2+^]_*i*_ in cystic cells (Mekahli et al., 2013; Yamaguchi et al., 2004, 2006). Of note, the Ca^2+^ permeable PC2 channel lacks intrinsic mechanosensitive properties, but it can heteromerize with TRPV4 to produce a functional mechanoactivated channel in renal epithelium (Du et al., 2012; Kottgen et al., 2008; Zhang et al., 2013). While this strongly indicates that deficient TRPV4 activity can lead to PKD pathology, its deletion, somewhat surprisingly, does not cause renal cystic phenotype in mice and zebra fish (Kottgen et al., 2008). On the contrary, we showed an impaired TRPV4‐dependent Ca^2+^ influx and deficient channel glycosylation in freshly isolated monolayers of cystic cells of PCK453 rats, a homologous model of human ARPKD (Zaika et al., 2013), and in primary cultured cystic cells of human ADPKD kidneys (Tomilin et al., 2018) pointing to a common underlying mechanism associated with channel dysfunction in driving cystogenesis. Moreover, TRPV4 agonist, GSK1016790A, was capable of reducing cystogenesis in PCK453 rats (Zaika et al., 2013). Overall, this argues that the systemic factors leading to TRPV4 stimulation might be of clinical relevance in counteracting PKD progression.

It has been recently shown that TRPV4‐mediated Ca^2+^ influx is the critical adaptive mechanism in promoting kaliuresis upon dietary K^+^ load (Pyrshev et al., 2022). Increased dietary K^+^ intake leads to a marked upregulation of renal TRPV4 expression and activity to facilitate flow‐induced K^+^ secretion via large conductance Ca^2+^ activated BK channel in the collecting duct (Mamenko et al., 2017). To this end, the current investigation aimed to determine how physiologically relevant manipulation of TRPV4 activity and expression by dietary K^+^ regimen affects cAMP signaling at the cellular level and cystogenesis in PCK453 rats.

## 2 MATERIALS AND METHODS

### 2.1 Reagents

All chemicals and materials were from Sigma, VWR (Radnor), Fisher, and Tocris unless noted otherwise and were at least of reagent grade.

### 2.2 Research animals

A homologous human ARPKD animal model, PCK453 rats were originally purchased from Charles Rivers Laboratories and bred in local facility. One‐month‐old littermates (males and females in equal quantities) were randomly assigned to consume regular diet (0.9% K^+^, TD7012), high KCl diet (5% K^+^, TD150699), and high KBicarbonate/Citrate (KB/C) diet (5% K^+^ with Bicarbonate to citrate in 4:1 ratio, TD150759) for 1 month. All diets were purchased from Envigo. Animals were given either tap water ad libitum, or water containing 28 μg/kgBW of a TRPV4 inhibitor, GSK2193874 (estimated final concentration 40 nM); or 26 μg/kgBW of a TRPV4 activator, GSK1016790A (estimated final concentration 40 nM), as necessary for experimental design. Spot urine samples were collected at the last day of the respective treatments before sacrifice between 10 am and 11 am to minimize contribution of the circadian cycle. Cardiac blood punctures were done post mortem. Previous characterization of PCK453 rats showed comparable kidney‐to‐total bodyweight ratio and cystic volume in males and females at 8 weeks, as used in this study, with significantly accelerated ARPKD progression being observed in males starting from 18 weeks (Mason et al., 2010).

### 2.3 Systemic measurements

Urinary and plasma K^+^ concentrations were measured using Jenway PFP7 Flame photometer (Bibby Scientific). Plasma was separated by centrifugation at 1300 g in Vacutainer Plus SST plastic tubes with clot activator and gel for plasma separation (BD; Cat. # 367988). Urinary pH was measured using MI‐410 pH microelectrode (Microelectrodes Inc.). Urinary creatinine concentration was assessed with QuantiChrom Creatinine Assay Kit (BioAssay Systems; Cat. # DICT‐500) utilizing improved Jaffe method (Mamenko et al., 2012). Aldosterone was measured using an enzymatic immunoassay kit (Cayman Chemical; Cat. # 501090) in accordance with the vendor's protocol. Kidney injury marker 1 (KIM1) was measured using a commercially available kit (R&D System; Cat. # RKM100).

### 2.4 Histological analysis

Kidneys from PCK453 rats were paraffin embedded using the standard protocols and cut into 5 μm thick sections around central area. Trichrome staining kit (Abcam, Cat. # ab150686) was applied for visualization of overall morphology, as well as collagenous connective tissue fibers in kidney sections. Two maximally distal sections within the same kidney from at least 4 animals were used for analysis. Images were quantified using ImageJ 1.50 software (NIH) to calculate cystic and fibrotic areas. For quantification of cystogenesis, the RGB images of the kidney sections were converted to the monochrome binary bit depth, using the Threshold tool. Particle Analyzer tool was applied to the processed images for calculating the number and the area of large cysts (>2 mm^2^ area) and small dilations (0.2–2 mm^2^ area) separately. For renal fibrosis area calculations, blue color was extracted from the RGB images of the kidney sections with calculations being performed as similarly described above. Renal fibrosis was presented as a percentage of the total area of the renal section.

### 2.5 Immunofluorescent microscopy

Paraffin embedded 5 μm thick kidney sections from PCK453 rats were used for analysis. For deparaffinization and fixation, the samples were subsequently washed with xylene (3 times for 5 min), 100% (2 times for 1 min), 95% (1 min), 70% (1 min) ethanol, and finally distilled water (5 min). The samples were further treated with antigen retrieval solution (BD Pharmingen Retrievagen A, pH 6.0; Fisher Scientific; Cat. # BDB550524) two times for 3 min. After extensive washout, kidney sections were permeabilized with 0.1% Triton ×100 (Sigma‐Aldrich; Cat. # 56H0850) for 10 min and treated with 10% normal goat serum for an hour at room temperature. Sections were incubated overnight at +4°C with anti‐AQP2‐ATTO Fluor‐550 (1:200, Alomone Labs; Cat. # AQP2‐002‐AO) and anti‐TRPV4 (1:500, Alomone labs; Cat.#. ACC‐034) antibodies. Followed by washing with PBS for 20 min at room temperature, the samples were incubated with Alexa 488 F(ab') secondary antibodies (1:1000 Invitrogen, Eugene, OR, USA; Cat. # A1182668). After washing with PBS for 20 min at room temperature, nuclei were stained with DAPI (0.5 μg/mL) for 10 min at room temperature. The samples were mounted with Fluoromount mounting media (Thermo Scientific). The labeled kidney sections were imaged with a Nikon A1R confocal microscope, as we did similarly before (Berrout et al., 2012; Mamenko et al., 2016). In brief, samples were excited with 405, 488, and/or 561 nm laser diodes and emission captured with a 16‐bit Cool SNAP HQ^2^ camera (Photometrics) interfaced to a PC running NIS elements software.

### 2.6 Western blotting

Freshly isolated kidneys were placed on ice, decapsulated, and homogenized in 3 volumes of ice‐cold lysis buffer containing 50 mM TrisCl, 5 mM EDTA and 1% Triton X‐100 (pH 7.5) supplemented with Complete Mini protease and PhosSTOP phosphatase inhibitor cocktails (Roche Diagnostics). The homogenates were centrifuged at 1000*g* for 15 min at +4°C, and sediment was discarded. Protein concentration was determined with NanoPhotometer N60 by a standard absorbance protocol. The samples (40 μg/lane) were separated on 9% polyacrylamide gels at 150 V for 90 min and transferred to a nitrocellulose membrane for 1.5 h at 100 V. Nitrocellulose membranes were incubated with primary anti‐TRPV4 antibodies (1:1000, Alomone labs; Cat.#. ACC‐034) overnight at +4 °C. Upon washout (3 times for 10 min in TBS‐Tween), the membrane was incubated with peroxidase‐conjugated goat anti‐rabbit (1:10000, Jackson ImmunoResearch Laboratories, Cat. # NC9448271) secondary antibodies for 1 h at room temperature. Ponceau red staining was used to verify equal protein load in different samples. Blots were quantified using ImageJ 1.50 software (NIH). The intensities of the non‐glycosylated (lower) and glycosylated (upper) bands were normalized to the total signal of the respective line in Ponceau red staining.

### 2.7 Mechanical isolation of collecting duct derived cystic cell monolayers

Kidneys were decapsulated and sliced into 2 mm thick sections. Monolayers of cells were mechanically isolated from open cyst cavities under stereomicroscope using watchmaker forceps, as we described previously (Pavlov et al., 2015; Zaika et al., 2013). The isolated monolayers were attached apical side upward to square 5 × 5 mm cover‐glasses coated with poly‐L‐lysine and placed into ice‐cold physiologic saline solution buffered with HEPES (pH 7.35). The monolayers were used within 2 h of isolation.

### 2.8 Intracellular Ca^2+^ measurements

Monolayers of cystic cells were loaded with Fura‐2 by incubation with 2 μM Fura‐2/AM acetoxymethyl ester in the bath solution for 40 min at room temperature followed by a washout with bath solution for additional 10 min. Cover glasses with cystic monolayers were placed in an open‐top imaging study chamber (RC‐26GLP; Warner Instruments) with a bottom coverslip viewing window and the chamber attached to the microscope stage of a Nikon Ti‐S Wide‐Field Fluorescence Imaging System (Nikon Instruments) integrated with Lambda XL light source (Sutter Instrument) and QIClick 1.4 megapixel monochrome CCD camera (QImaging) via NIS Elements 4.3 Imaging Software (Nikon Instruments). Cells were imaged with a 40× Nikon Super Fluor objective, and regions of interest (ROIs) were drawn for individual cells. For [Ca^2+^]_*i*_ measurements, the Fura‐2 fluorescence intensity ratio was determined by excitation at 340 and 380 nm and calculating the ratio of the emission intensities at 511 nm in the usual manner every 5 s. The changes in 340/380 ratio were converted into changes in intracellular calcium concentration, as we described in great details previously (Mamenko, Zaika, O'Neil, et al., 2013). Experiments were performed under permanent perfusion of a bath solution containing (in mM): 150 NaCl, 5 KCl, 1 CaCl_2_, 2 MgCl_2_, 5 glucose, and 10 HEPES at 1.5 mL/min rate. For mechanical stimulation, an abrupt increase of perfusion flow to 15 mL/min (10×) was used. This produces a shear stress of 3 dyne/cm^2^, which is within the physiological range existing in the collecting duct in conditions with increased flow delivery, such as high K^+^ diet (Berrout et al., 2012). In average, 4 cystic cell monolayers (30–50 cells in each) from at least 3 different rats were used for each tested condition.

### 2.9 Statistical analysis

All summarized data are reported as mean ± SD. Statistical comparisons were made using one‐way ANOVA with post hoc Tukey test or one‐way repeated ANOVA with post hoc Bonferroni test (for paired experiments within the same group). *p* value <0.05 was considered significant.

## 3 RESULTS

### 3.1 Elevated K^+^ intake increases TRPV4 expression in the kidney of ARPKD PCK453 rats

Abundant experimental evidence demonstrates a central role of Ca^2+^‐permeable TRPV4 channel in flow‐sensitive [Ca^2+^]_*i*_ signaling of the renal tubule (Berrout et al., 2012; Mamenko et al., 2017; Pyrshev et al., 2022). TRPV4 activity is drastically impaired in cyst cells of ARPKD (Zaika et al., 2013) and ADPKD (Tomilin et al., 2018). Here, we aimed to examine whether physiologically relevant stimulation of TRPV4 expression and activity is capable of reducing cystogenesis. To this end, we fed PCK453 rats with either high KCl or high K organic anion (bicarbonate: citrate, B/C as 4:1) diets for 1 month to also investigate a role of accompanying anions. Of note, theses diets contained the same amount of K^+^ (approximately 5%). Neither of the treatments induced notable adverse alterations in systemic K^+^ balance. Thus, both high K^+^ diets did not affect plasma K^+^ levels, when compared to the control group (Figure S1a). Aldosterone levels were similarly increased in high KCl and high KB/C groups (Figure S1b), although relative urinary K^+^ excretion, defined as K^+^/creatinine ratio, was somewhat greater in high KCl group (Figure S1c). Urinary pH was more acidic in high KCl group and more alkalic in high KB/C group, as expected (Figure S1d).

**FIGURE 1:**
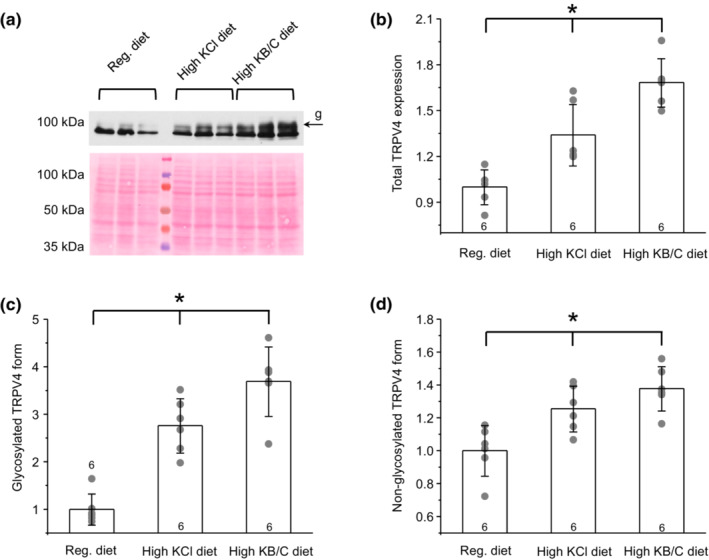
High KCl and high KB/C (bicarbonate/citrate) diet increases renal TRPV4 expression in ARPKD PCK453 rats. (a) Representative Western blot probed with anti‐TRPV4 antibodies from whole kidney lysates of PCK453 rats kept on regular (0.9%K^+^), high KCl (5%K^+^), and high KB/C (5%K^+^, bicarbonate: citrate as 4:1) diets for 1 month. Each line represents individual animal. The Ponceau red staining of the same nitrocellulose membrane demonstrating equal protein loading is shown below. “g” denotes upper glycosylated form of TRPV4. Summary graphs comparing total (b) glycosylated (c) and non‐glycosylated (d) forms of TRPV4 levels in PCK453 rats from the conditions in (a). The intensity values were normalized to the total signal of the respective lines in Ponceau red staining. Data are presented as mean ± SD. Numbers of each experimental groups (individual animals) are shown below. *Significant changes (*p* < 0.05, one‐way ANOVA with post hoc Tukey test) between experimental groups shown with brackets on the top.

As shown on the representative Western blot from whole kidney homogenates (Figure 1a) and the summary graph (Figure 1b), both diets significantly increased renal TRPV4 expression with the stimulatory effect being more pronounced in high KB/C group. It is important to note that TRPV4 appears as a duplet of bands with higher representing a heavy mannitol sugar glycosylation form (Wu et al., 2007; Xu et al., 2006). Drastically decreased TRPV4 glycosylation was observed during both ARPKD and ADPKD (Tomilin et al., 2018; Zaika et al., 2013). Interestingly, both high KCl and high KB/C diets led to a much stronger upregulation of the glycosylated (Figure 1c) than non‐glycosylated (Figure 1d) forms. Again, the effect was considerably stronger in high KB/C‐treated group.

We next explored the underlying cause of a higher stimulatory effect of high KB/C diet on renal TRPV4 expression in PCK453 rats. For this, we monitored the specific sites of TRPV4 expression with immunofluorescent microscopy in the renal sections from PCK453 rats fed control, high KCl, and high KB/C diets. Since over 98% of the renal tubules do not undergo transformation into cysts/dilations (Bergmann et al., 2018), their contribution to the overall renal TRPV4 expression (Figure 1a) will be dominant. TRPV4 was primarily localized to the apical and subapical regions of AQP2‐positive collecting ducts in control (Figure S2). We observed a very similar pattern in high KCl treated rats with TRPV4‐reporting signal being stronger using the same laser settings (Figure S2). Interestingly, we detected accumulation of the TRPV4‐reporting signal on both apical and basolateral sides of AQP2‐positive collecting ducts, as well as notable appearance of TRPV4 in AQP2‐negative cortical tubules (most likely proximal segments) on the basolateral side in rats fed high KB/C diet (Figure S2). We concluded that the increased basolateral TRPV4 presence in both collecting ducts and the proximal tubule explains the overall higher renal TRPV4 expression in high KB/C versus high KCl groups.

### 3.2 High KCl and KB/C diets elicit opposite effects on TRPV4 activity in cyst cells of PCK453 rats

**FIGURE 2:**
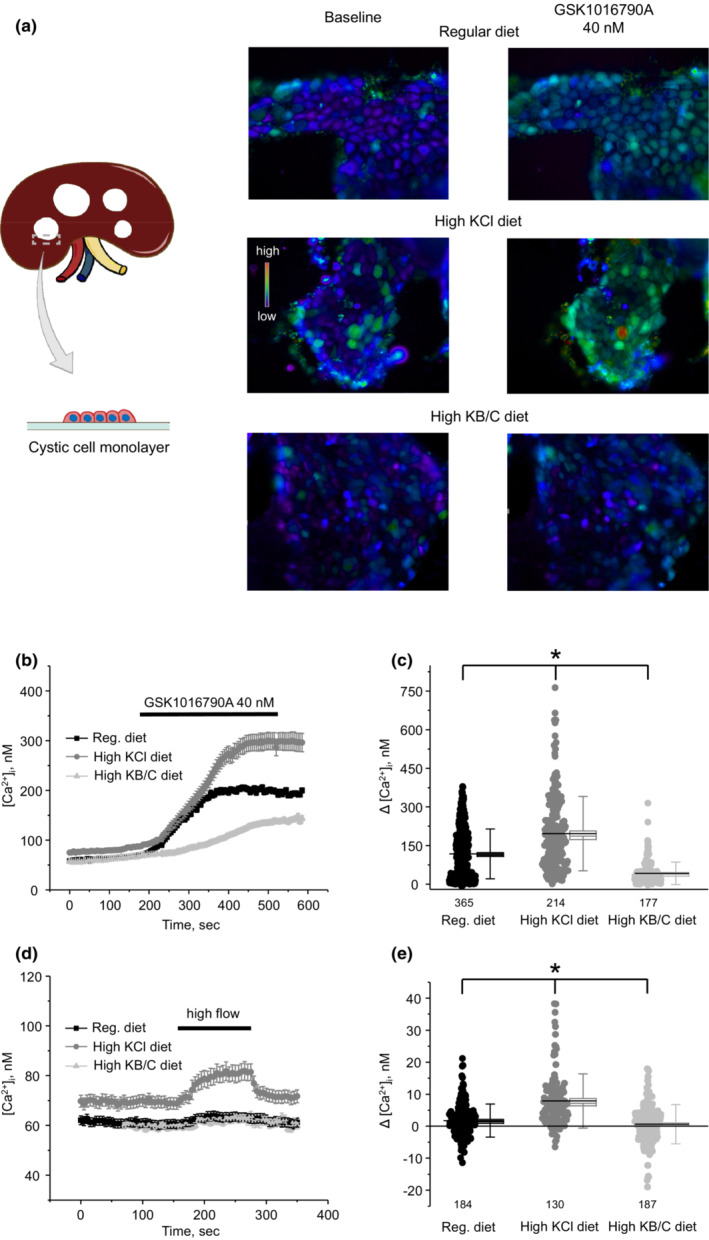
TRPV4‐dependent Ca^2+^ influx and flow‐induced [Ca^2+^]_*i*_ signaling in cystic cells are stimulated by KCl but inhibited by high KB/C diet. (a) Shown on left is schematic representation of isolation of cystic cell monolayers using watchmaker forceps from kidneys of PCK453 rats suitable for [Ca^2+^]_*i*_ imaging. Representative pseudocolor images of [Ca^2+^]_*i*_ changes (blue—low and red—high) in freshly isolated cystic cell monolayers loaded with Ca^2+^‐sensitive dye Fura2 at the baseline and following 5 min application of 40 nM TRPV4 agonist, GSK1016790A from PCK453 rats kept on regular (0.9%K^+^), high KCl (5%K^+^), and high KB/C (5%K^+^, bicarbonate: citrate as 4:1) diets for 1 month. (b) The averaged time courses of [Ca^2+^]_*i*_ changes upon application of 40 nM GSK1016790A (shown with the bar on top) in individual cystic cells within monolayer for the conditions in (a). The numbers of individual cells are shown. The data were obtained from at least 4 different monolayers isolated from at least 3 different rats for each group. Individual analysis for each tested monolayer is shown in Figure S3. (c) Summary graph comparing the magnitudes of GSK1016790A‐mediated [Ca^2+^]_*i*_ elevations calculated as the difference in [Ca^2+^]_*i*_ values before and after application of the TRPV4 agonist in individual cystic cells from the conditions in (a). The numbers of individual cells are shown. Bars and whiskers represent SE and SD, respectively. Mean and median values are denoted with lines (long and short). *Significant changes (*p* < 0.05, one‐way ANOVA with post hoc Tukey test) between experimental groups shown with brackets on the top. (d) The averaged time courses of [Ca^2+^]_*i*_ changes upon abrupt increase in perfusion flow from 1.5 mL/min to 15 mL/min (shown with the bar on top) in individual cystic cells within monolayer for the conditions in (a). (e) Summary graph comparing the magnitudes of flow‐induced [Ca^2+^]_*i*_ elevations calculated as the difference in [Ca^2+^]_*i*_ values before and at the end of high flow application in individual cystic cells from the conditions in (a). The numbers of individual cells are shown. Bars and whiskers represent SE and SD, respectively. Mean and median values are denoted with lines (long and short). *Significant changes (*p* < 0.05, one‐way ANOVA with post hoc Tukey test) between experimental groups shown with brackets on the top.

While the observed upregulation of TRPV4 channel by dietary K^+^ supplementation is promising, it is imperative to augment channel activity to restore normal [Ca^2+^]_*i*_ homeostasis in cystic cells. We have previously developed a method of mechanical isolation of cystic cell monolayers suitable for [Ca^2+^]_*i*_ imaging from open cyst cavities in kidneys of PCK453 rats (Zaika et al., 2013). Thus, we next directly monitored TRPV4‐dependent Ca^2+^ influx upon application of a highly potent and selective channel agonist, GSK1016790A in freshly isolated cystic cells from animals fed control, high KCl, and high KB/C diets for 1 month. We showed previously that GSK1016790A has no effect on [Ca^2+^]_*i*_ when TRPV4 was deleted (Berrout et al., 2012; Mamenko et al., 2017), implying that the agonist‐induced elevations in [Ca^2+^]_*i*_ are indeed TRPV4‐dependent. Representative micrographs of [Ca^2+^]_*i*_ before and following application of GSK1016790A (40 nM for 5 min) are shown in Figure 2a, and the respective time courses of [Ca^2+^]_*i*_ changes for each condition are shown in Figure 2b. Figure S3 shows no apparent heterogeneity in [Ca^2+^]_*i*_ responses in individual monolayers of cystic cells for all tested conditions. As summarized in Figure 2c, GSK1016790A induced moderate increases of [Ca^2+^]_*i*_ by 117 ± 96 nM (*n* = 366 cells, *N* = 5 kidneys) in cystic cells from the control diet group. These values were significantly increased to 196 ± 144 nM (*n* = 214 cells, *N* = 4 kidneys; *p* < 0.05) in PCK453 rats fed high KCl. Unexpectedly, we observed much reduced GSK1016790A‐induced [Ca^2+^]_*i*_ responses in cystic cells from high KB/C group: 42 ± 43 nM (*n* = 177 cells, *N* = 4 kidneys; *p* < 0.05). We have also stimulated TRPV4 by increasing fluid flow over the apical plasma membrane from 1.5 mL/min to 15 mL/min, as we similarly did before (Mamenko et al., 2017; Zaika et al., 2013). As shown on the averaged time course (Figure 2d) and the summary graph of the amplitude of flow‐induced [Ca^2+^]_*i*_ elevations (Figure 2e), cystic cells only marginally responded to high flow in the control group and the effect was notably stronger in high KCl group, whereas no significant response to flow was detected in cystic cells from high KB/C group. Moreover, basal [Ca^2+^]_*i*_ levels, another index of TRPV4 activity (Tomilin et al., 2018), were increased in high KCl diet versus control and high B/C diets: 73 ± 23 nM (*n* = 214 cells, *N* = 4 kidneys), 59 ± 20 nM (*n* = 366 cells, *N* = 5 kidneys; *p* < 0.05), and 55 ± 20 nM (*n* = 177 cells, *N* = 4 kidneys; *p* < 0.05), respectively.

Overall, our current results show that while both high KCl and high KB/C diets augment renal TRPV4 expression, high KCl increases TRPV4 activity, whereas paradoxically high KB/C decreases channel activity in cystic cells of PCK453 rats.

### 3.3 TRPV4 activity in cystic cells exhibits inverse correlation with cystogenesis and renal injury marker in PCK453 rats

**FIGURE 3:**
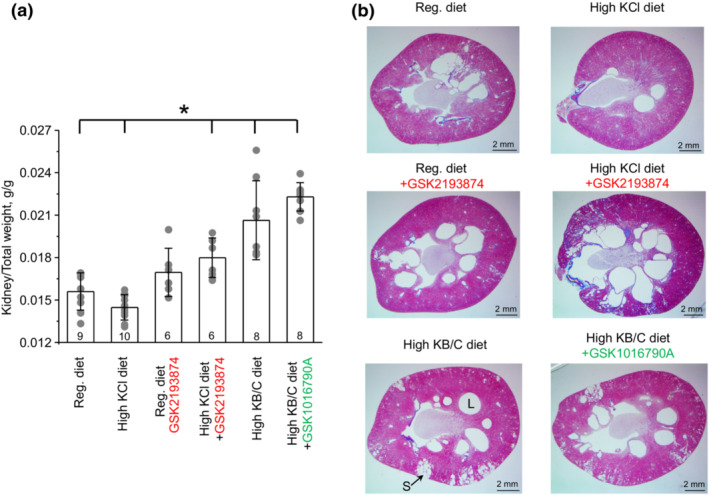
Regulation of TRPV4 activity by K^+^ intake affects renal morphology in PCK453 rats. (a) Summary graph of the kidney‐to‐total bodyweight ratio in PCK453 rats fed regular (0.9%K^+^), high KCl (5%K^+^) diets in the absence and presence of TRPV4 antagonist, GSK2193874 (highlighted in red) in drinking water (28 μg/kgBW, estimated on‐cell concentration of 40 nM), high KB/C (5%K^+^, bicarbonate: citrate as 4:1) diet, and high KB/C with TRPV4 activator, GSK1016790A (highlighted in green) in drinking water (26 μg/kgBW, estimated on‐cell concentration of 40 nM) for 1 month. Data are presented as mean ± SD. Numbers of each experimental groups (individual animals) are shown below. *Significant changes (*p* < 0.05, one‐way ANOVA with post hoc Tukey test) versus regular diet as showed with brackets on the top. (b) Representative kidney sections of PCK453 rats from the conditions in A processed with trichrome staining kit to quantify morphology/cystogenesis and collagen fiber accumulation (fibrosis, blue color). “L” represents a large cyst, whereas “S” shows small cystic dilations.

We next investigated how augmented TRPV4 activity in cystic cells during high KCl diet and impaired TRPV4 activity during high KB/C diet contributes to ARPKD progression at the whole kidney level. As summarized in Figure 3a, 1 month treatment with high KCl diet significantly decreased kidney to total bodyweight ratio indicative of reduced cystogenesis, when compared to the control group fed regular diet. To elucidate a potential role of TRPV4, PCK453 rats were similarly fed with either regular or high KCl diet on the background of an orally active TRPV4 antagonist, GSK2193874 (28 μg/kgBW). Systemic TRPV4 blockade had a tendency to increase kidney‐to‐total bodyweight ratio during regular diet versus control (Figure 3a). Importantly, the beneficial effect of high KCl diet was reversed upon concomitant treatment with GSK2193874 leading to increased kidney‐to‐total bodyweight ratio. Similarly, 1‐month treatment with high KB/C diet (low TRPV4 activity in cystic cells) led to a significant increase in kidney‐to‐total bodyweight ratio, suggesting an accelerated cystogenesis (Figure 3a). Of note, systemic administration of TRPV4 agonist, GSK1016790A (26 mg/kgBW) did not significantly change the ratio when compared to the condition of high KB/C diet. This is consistent with only a mild stimulatory effect of GSK1016790A on TRPV4‐dependent Ca^2+^ influx in cystic cells shown in Figure 2. Overall, these results support the notion that the effects of dietary K^+^ supplementation on reduction in the kidney‐to‐bodyweight ratio depend on the magnitude of TRPV4 activity in cystic cells but not on total TRPV4 expression in PCK453 rats. It is also important to mention that the prolonged consumption of either high KCl or high KB/C led to similar decreases in total bodyweight of the tested animals compared to that kept on regular diet (Figure S4). In turn, this would lead to underestimation of the high KCl condition due to the considerably lower denominator in the ratio. At the same time, neither GSK2193874 (TRPV4 blocker) nor GSK1016790A (TRPV4 activator) had a significant effect on total bodyweight (not shown).

**FIGURE 4:**
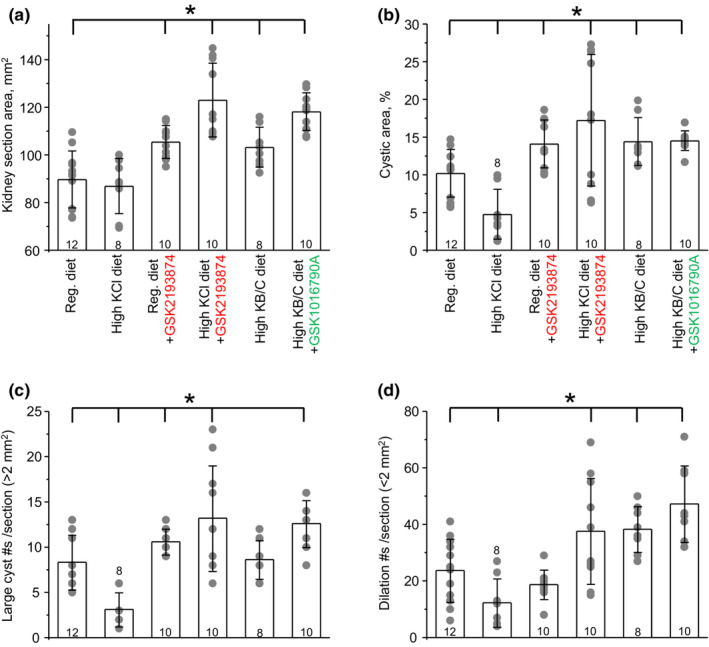
TRPV4 activity inversely correlates with renal cystogenesis in PCK453 rats. Summary graphs of total kidney section area (a), cystic area (b), number of large cysts per section (c), and number of small dilations per section (d) in PCK453 rats fed regular (0.9%K^+^), high KCl (5%K^+^) diets in the absence and presence of TRPV4 antagonist, GSK2193874 (highlighted in red) in drinking water (28 μg/kgBW, estimated on‐cell concentration of 40 nM), high KB/C (5%K^+^, bicarbonate: citrate as 4:1) diet, and high KB/C with TRPV4 activator, GSK1016790A (highlighted in green) in drinking water (26 μg/kgBW, estimated on‐cell concentration of 40 nM) for 1 month. Data are presented as mean ± SD. Numbers of each experimental groups are shown below. Kidney sections from at least 4 different animals were used for each tested group. *Significant changes (*p* < 0.05, one‐way ANOVA with post hoc Tukey test) versus regular diet as shown with brackets on the top.

**FIGURE 5:**
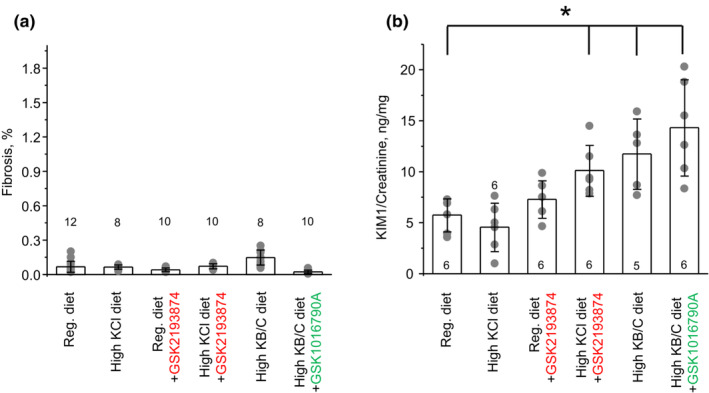
Impaired TRPV4 activity correlates with increased renal injury marker in PCK453 rats. Summary graphs of percent of fibrotic area per kidney section similar to shown in Figure 3b (a) and KIM1 to creatinine ratio in urine (b) in PCK453 rats fed regular (0.9%K^+^), high KCl (5%K^+^) diets in the absence and presence of TRPV4 antagonist, GSK2193874 (highlighted in red) in drinking water (28 μg/kgBW, estimated on‐cell concentration of 40 nM), high KB/C (5%K^+^, bicarbonate: citrate as 4:1) diet, and high KB/C with TRPV4 activator, GSK1016790A (highlighted in green) in drinking water (26 μg/kgBW, estimated on‐cell concentration of 40 nM) for 1 month. Data are presented as mean ± SD. Numbers of each experimental groups (kidneys or urinary samples from different animals) are shown below. *Significant changes (*p* < 0.05, one‐way ANOVA with post hoc Tukey test) versus regular diet as shown with brackets on the top.

We further assessed how interventions shown in Figure 3a affect renal cystogenesis, using renal sections from PCK453 rats. As shown in Figure 3b, large cysts were visible primarily in the medullary area, whereas much smaller dilations were rarely present in the medulla and cortex of control group, as expected for 2 months old PCK453 rats. All cysts were positive for AQP2 (see further), which is consistent with their collecting duct origin, as is expected for ARPKD (Bergmann et al., 2018). Figure 4 contains quantitative assessment of the total kidney section area (Panel A), cystic area (Panel B), the number of large (area > 2 mm^2^) cysts per section (Panel C), and small (0.2 mm^2^ < area < 2 mm^2^) dilations (Panel D). Renal sections from animals on high KCl diet had notably fewer cysts (Figure 3b). Specifically, the cystic area was reduced from 10.2% ± 3.1% in control group to 4.7% ± 3.3% upon KCl (*p* = 0.002; Figure 4b). This was attributed to the significantly decreased number of large cysts (Figure 4c) and small dilations (Figure 4d). In contrast, inhibition of TRPV4 with GSK2193874 markedly augmented cystogenesis similarly on both regular and high KCl diet (Figure 3b). Thus, the cystic areas were increased to 14.1% ± 3.1% (*p* = 0.009) and 17.2% ± 8.7% (*p* = 0.016) in GSK2193874 and high KCl + GSK2193874 groups, respectively. The total kidney section areas were also significantly increased in both groups (Figure 4a). The average number of large cysts was increased in GSK2193874 and high KCl + GSK2193874 (Figure 4c), whereas the number of small dilations was augmented only in renal sections from KCl + GSK2193874 group (Figure 4d). Similarly, high KB/C diet alone and in combination with GSK1016790A led to a significantly greater cystogenesis with cystic area being 14.4% ± 3.1% (*p* = 0.009) and 14.5% ± 1.3% (*p* = 0.0006), respectively (Figure 4b). Most notably, this was accompanied with the appearance of a large number of small dilations in the cortical area (Figure 3b and 4d). As shown in Figure S5, these dilations were AQP2‐positive suggesting that they were developed from the cortical collecting ducts and/or connecting tubules and not from other tubular segments. Despite apparent cystogenesis, the extent of renal fibrosis (assessed using Trichrome staining kit, as exemplified in Figure 3b) was minimal (<0.3% of the total area) in all tested groups (Figure 5a). This is consistent with fibrosis being significant in 18 week old PCK453 rats and much less in 8 weeks, as used in the current study (Mason et al., 2010). However, urinary KIM1 levels were significantly elevated in high KCl + GSK2193874, high KB/C, and high KB/C + GSK1016790A groups (Figure 5b) pointing to an augmented renal injury in the conditions associated with low TRPV4 activity.

### 3.4 Functional TRPV4 activity controls cAMP levels in cystic cells

**FIGURE 6:**
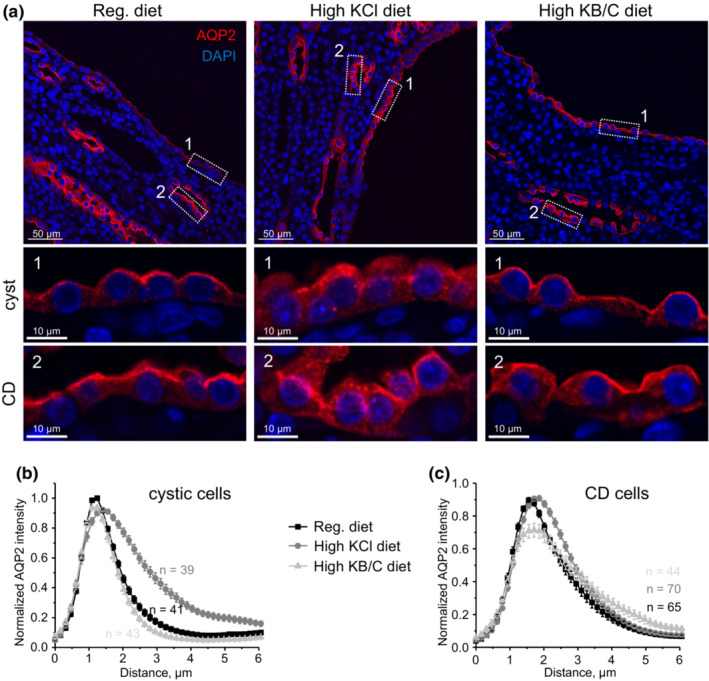
TRPV4 activity regulates subcellular AQP2 distribution in cystic cells of PCK453 rats. (a) Representative confocal images showing AQP2 (pseudocolor red) distribution in kidney sections of PCK453 rats fed regular (0.9%K^+^), high KCl (5%K^+^), and high KB/C (5%K^+^, bicarbonate: citrate as 4:1) diets for 1 month. Nuclear Dapi staining is shown with pseudocolor blue. Areas with cystic (1) and non‐dilated collecting duct (2) are shown below at higher magnification. The averaged intensities of AQP2‐reporting fluorescent signals around the apical area in cystic (b) and non‐dilated collecting duct (c) cells from the conditions in (a). For each individual cell the fluorescent signals were normalized to their corresponding maximal value.

**FIGURE 7:**
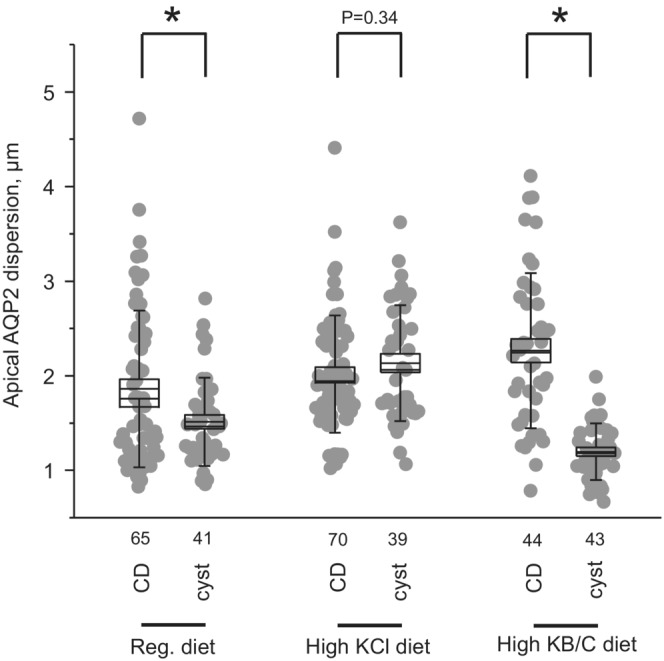
TRPV4 activity is inversely related to cAMP levels in cystic cells of PCK453 rats. Summary graph comparing dispersion (decrease by 50% from maximum) of AQP2‐reporting signal in non‐dilated collecting duct versus cystic cells in kidney sections of PCK453 rats fed regular (0.9%K^+^), high KCl (5%K^+^), and high KB/C (5%K^+^, bicarbonate: citrate as 4:1) diets for 1 month. Bars and whiskers represent SE and SD, respectively. Mean and median values are denoted with lines. Numbers of each experimental groups are shown below. Kidney sections from at least 4 different animals were used for each tested group. *Significant changes (*p* < 0.05, one‐way ANOVA with post hoc Tukey test) between groups shown with brackets on the top.

We next explored the mechanism of inversed relation between TRPV4 activity and renal cystogenesis in PCK453 rats. The compelling accumulated evidence demonstrates the central role of augmented cAMP levels in driving epithelial cell transformation to malicious cystic phenotype and enhanced proliferation in a B‐Raf/ERK‐dependent manner (Belibi et al., 2004; Wallace, 2011; Yamaguchi et al., 2003, 2006). Thus, we monitored subcellular AQP2 distribution, as an index of cAMP levels, in cystic cells with immunofluorescent microscopy in renal sections from control, high KCl (increased TRPV4 activity), and high KB/C (impaired TRPV4 activity) fed PCK453 rats (Figure 6a). As shown in the representative high magnification images and the respective line‐scan analysis of the intensity of AQP2‐reporting fluorescent signal, the maximum is observed in the apical and subapical regions of cystic cells from control animals pointing to elevated cAMP levels, known to promote apical AQP2 translocation (Figure 6b). Treatment with KCl diet resulted in apparent intracellular retention of AQP2 suggesting lower cAMP levels with the dispersion (decrease by 50% from maximum) being significantly increased from 1.51 ± 0.46 to 2.13 ± 0.61 μm (*p* < 0.05) in control and high KCl conditions, respectively (Figure 7). In contrast, even stronger accumulation of AQP2‐reporting signal at the apical membrane was seen in rats fed high KB/C diet (Figure 6b). The dispersion was significantly reduced to 1.19 ± 0.29 μm (*p* < 0.05) versus control condition pointing to further elevations in cAMP levels in this case (Figure 7). In contrast, line‐scan analysis of non‐dilated collecting ducts in the same sections showed comparable more diffuse intracellular distribution of the AQP2‐reporting signal in all tested conditions (Figure 6c). The dispersion values were 1.86 ± 0.82, 2.02 ± 0.61, and 2.28 ± 0.82 μm for control, high KCl, and high KB/C, respectively. As shown in the summary graph in Figure 7, there were significant differences in AQP2 distribution between non‐dilated collecting duct and cystic cells in control, which was further exacerbated in KB/C condition. In contrast, the distribution of AQP2‐reporting signal was very similar between non‐transformed collecting duct and cystic cells in high KCl treated PCK453 rats, which is consistent with reduced cAMP levels when TRPV4 activity is higher. Overall, our results in Figures 6 and 7 demonstrate an inversed correlation between TRPV4 activity and cAMP‐dependent AQP2 translocation in cystic cells which would explain accelerated/decelerated cystogenesis in PCK453 rats fed high KB/C and high KCl diets, respectively.

## 4 DISCUSSION

**FIGURE 8:**
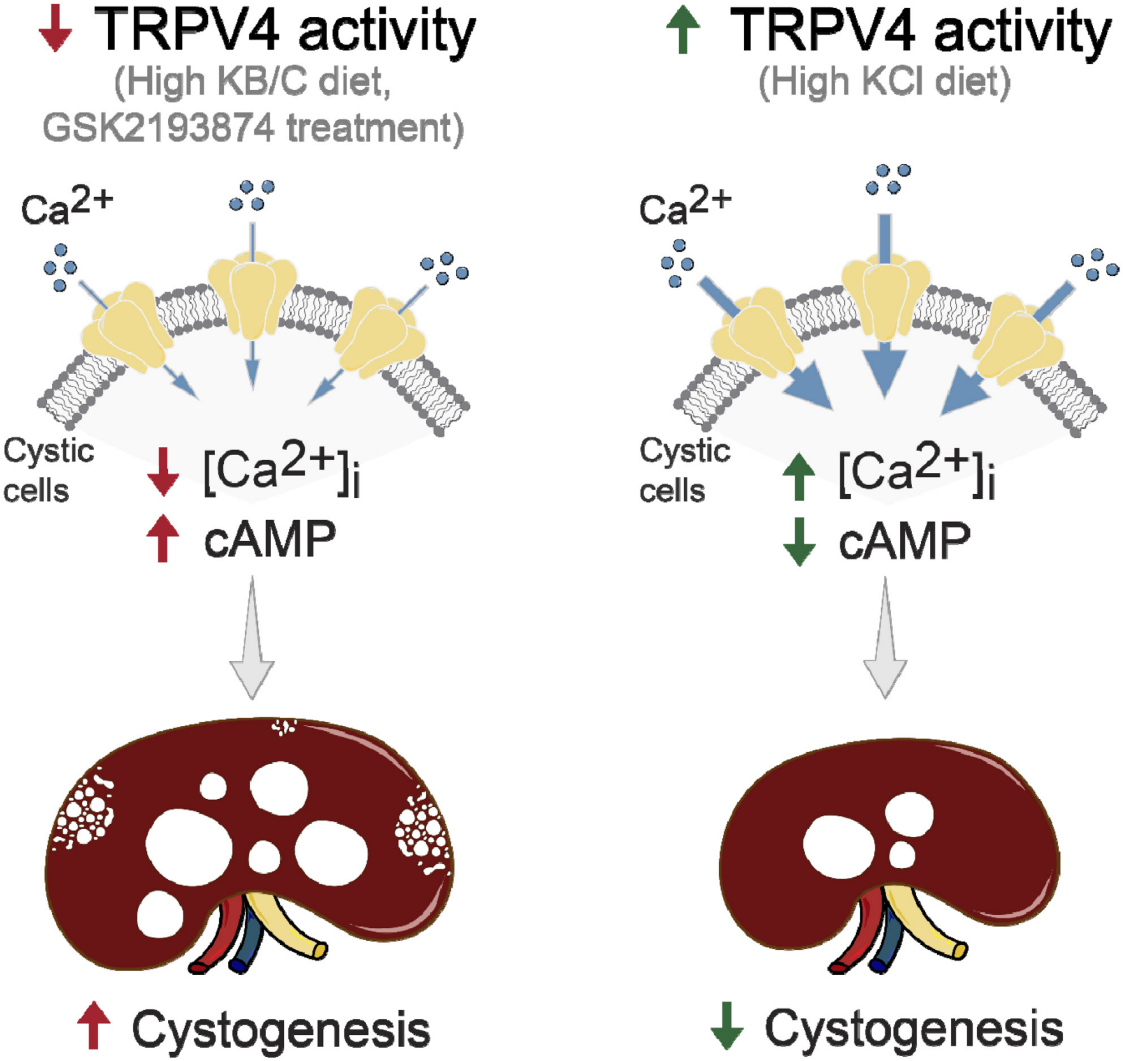
Principal scheme showing the significance of TRPV4 activity in cystic cells on ARPKD progression in PCK453 rats.

This study provides strong causal evidence of how functional activity of the mechanosensitive TRPV4 channel affects ARPKD progression at both cellular and whole kidney levels. Specifically, we demonstrate that physiologically relevant stimulation of TRPV4 expression and activity in cystic cells by high KCl diet increases basal [Ca^2+^]_*i*_ levels, partially restores mechanosensitivity, and causes intracellular AQP2 retention (reduced cAMP levels) to markedly slow the development and growth of renal cysts in PCK453 rats (Figure 8, right panel). In contrast, impaired TRPV4 activity in cystic cells either due to pharmacological antagonism with GSK2193874 or as a result of treatment with high KB/C diet favors the cAMP‐dependent phenotype thus greatly accelerating ARPKD progression and renal injury (Figure 8, left panel).

It is commonly accepted that PKD is associated with abnormally low basal [Ca^2+^]_*i*_ levels and inability to sense mechanical stimuli by cystic cells irrespective whether this occurs due to bending of the antenna‐like primary cilium (Nauli et al., 2003) or by an acute stretch of the plasma membrane (Delling et al., 2016). The disruption of the activity of multi‐protein complex, which includes atypical G‐protein coupled receptor PC1, Ca^2+^‐permeable channel PC2 (both mutated in ADPKD), and fibrocystin (mutated in ARPKD; Rossetti et al., 2007; Ward et al., 2002) reportedly accounts for the distorted [Ca^2+^]_*i*_ homeostasis in cystic cells (Bergmann et al., 2018; Ghata & Cowley, 2017). However, PC2 channel does not respond to mechanical stimulation (Du et al., 2012; Kottgen et al., 2008), which argues for a potential involvement of additional mechanosensitive components in the complex. Indeed, PC2‐TRPV4 functional hetero‐tetramers (presumably in 2:2 stoichiometry (Stewart et al., 2010)) have been reported in renal epithelial cells with their activity being compromised in cystic cells of both ADPKD and ARPKD (Tomilin et al., 2018; Zaika et al., 2013; Zhang et al., 2013). On the one hand, it is plausible to propose that the deficient mechanosensitive Ca^2+^ influx underlies a switch in the cellular responses to cAMP from anti‐proliferative in normal tubule cells to mitogenic signaling involving over‐activation of the via B‐Raf/ERK cascade in cystic cells (Gattone et al., 2003; Yamaguchi et al., 1997, 2004, 2006). On the contrary, genetic deletion of TRPV4 abolishes flow‐induced [Ca^2+^]_*i*_ responses and decreases basal [Ca^2+^]_*i*_ levels but does not lead to cystogenesis (Berrout et al., 2012; Kottgen et al., 2008) suggesting that this is not a requirement for transformation to a cystic phenotype. Having said that, we show that stimulation of TRPV4 activity in cystic cells with high KCl diet (Figure 2) leads to a markedly diffused intracellular distribution of AQP2 indicating lower cAMP levels, when compared to that in control (Figure 6a,b). Moreover, reductions in TRPV4‐dependent Ca^2+^ influx induced by the high KB/C diet promote apical AQP2 accumulation (higher cAMP) in cystic cells. At the whole kidney level, this correlates with significantly decelerated and accelerated renal cystogenesis, respectively (Figures 3 and 4). Of note, we previously demonstrated that non‐dilated collecting ducts of PCK453 rats have nearly normal mechanosensitivity and intact TRPV4 activity (Zaika et al., 2013). Consistently, our line‐scan analysis shows a much broader (compared to the observed in cystic epithelium) intracellular AQP2 distribution in collecting ducts irrespective of the dietary regimen (Figure 6a,c). This strongly implies that TRPV4 activity specifically in cyst cells is a critical factor which determines the rate of cystogenesis. Indeed, we observed appearance of multiple small cortical dilations in PCK453 rats of 8 weeks old upon treatment with high KB/C diet or TRPV4 antagonist GSK2193874 (Figure 3b), which corresponds to the disease stage of considerably older (18–25 weeks) rats kept in standard conditions (Mason et al., 2010).

It is unlikely that the impaired TRPV4 function in cystic cells is associated with decreased TRPV4 expression. We have previously showed comparable levels of the channel in primary cultured human ADPKD and non‐ADPKD cells (Tomilin et al., 2018) and only a mild reduction of 20%–30% in the kidneys of ARPKD PKC453 versus control S/D rats (Zaika et al., 2013). This cannot account for the observed 50%–80% decreases in TRPV4 single channel activity and TRPV4‐mediated Ca^2+^ influx in cystic cells in both cases. Strikingly, a dramatic decrease in TRPV4 glycosylation has been detected during both ADPKD and ARPKD (Tomilin et al., 2018; Zaika et al., 2013). The post‐translational modification of TRPV4 with high mannose sugars occurs very close to the pore region (Wu et al., 2007; Xu et al., 2006), which can have an immediate (acute) effect on channel gating. Indeed, glycosylation deficient TRPV4 mutants exhibit lower basal activity and disrupted stimulation by mechanical stimuli (Lamande et al., 2011). Furthermore, pharmacological blockade of glycosylation with tunicamycin decreases TRPV4 activity to the levels observed in cystic ADPKD cells (Tomilin et al., 2018). Notably, both high KCl and high KB/C diets have much stronger stimulatory effect on the abundance of the glycosylated form of TRPV4 in the kidney of PCK453 rats (Figure 1). Since we detected an upregulation of TRPV4‐reporting signal with immunofluorescent microscopy not only in cysts but also in non‐dilated collecting ducts as well as in proximal tubules for high KB/C condition (Figure S2), it is quite possible that the increase in TRPV4 glycosylation is predominantly attributable to the normal (i.e., non‐cystic) epithelium during both diets (Figure 1). This would explain the puzzling reduction of TRPV4 activity in freshly isolated cystic monolayers from high KB/C treated PCK453 rats (Figure 2). Moreover, channel glycosylation could also be quite low in cystic cells from high KCl treated mice because the detected TRPV4‐dependent Ca^2+^ influx (Figure 2b) and mechanosensitive [Ca^2+^]_*i*_ responses (Figure 2d) are only at approximately 50% of the values existing in non‐dilated collecting ducts of PCK453 rats (Zaika et al., 2013). Currently, we do not know how high KB/C diet decreases TRPV4 activity in cystic cells. TRPV4 is known to have a relatively weak pH dependence with channel activity being stimulated by low extracellular pH (Mizuno et al., 2003). Thus, it is possible that high KB/C diet‐induced alkalization would exhibit an inhibitory effect on TRPV4.

We observed a much reduced TRPV4‐dependent Ca^2+^ influx in cystic cells from rats on high KB/C diet compared to controls (Figure 2) despite higher overall TRPV4 expression (Figure 1) and seeming membrane localization (Figure S2). This suggests that the channel is locked in non‐active (or low active) state with increased abundance representing a compensatory mechanism. In fact, we previously demonstrated that TRPV4 trafficking is upregulated by cAMP‐PKA mechanism in collecting duct cells (Mamenko, Zaika, Boukelmoune, et al., 2013), which is consistent with predominantly membrane localization of TRPV4 in cystic cells possessing high cAMP levels. However, this cAMP‐dependent TRPV4 translocation does not convert into augmented TRPV4‐dependent Ca^2+^ influx with channel requiring stimulation of protein kinase C to become active (Mamenko, Zaika, Boukelmoune, et al., 2013). Of interest, recent study demonstrates that expression of PKCζ isoform is downregulated in patients with autosomal dominant PKD with its restoration correlating with reduced cystogenesis in multiple PKD mouse models (Akbari et al., 2022). Thus, it is plausible to propose that this improvement could be at least partially TRPV4‐dependent. Further studies are necessary to test this hypothesis.

Increased dietary NaCl intake has been commonly associated with acceleration in PKD progression (Bergmann et al., 2018). Furthermore, treatment with both high and low NaCl diets led to an acceleration in renal cyst development in PCK453 rats (Ilatovskaya et al., 2019). Thus, the contrasting effects of NaCl and KCl diets on cystogenesis in the absence of any beneficial effect of changes in dietary Cl^−^ between low and high salt diets imply that the observed improvement in renal cystogenesis in PCK453 rats fed high KCl diet are driven by elevated K^+^. At the same time, the observation that high KB/C diet accelerates cystogenesis while high KCl diet reduces the development of cysts in PCK453 rats is somewhat surprising (Figure 3). The previously published evidence suggests that acidification and azotemia accelerates renal cyst growth in an ADPKD model, Han:SPRD rats (Cowley et al., 1996). In contrast, alkalization upon supplementation with either bicarbonate or citrate reported to protect against decline in GFR and to some extent reduced renal cystic burden in this model (Tanner & Tanner, 2000; Torres et al., 1994). In fact, we paired high K^+^ with both citrate and bicarbonate in this study in anticipation to obtain an additional improvement of renal function beyond that seen with high KCl diet. It turns out that our initial hypothesis led to opposite results with high KB/C treatment causing markedly larger kidney weight (Figure 3a) and number of cysts in renal sections (Figure 4) despite prominent alkalization of urinary pH (Figure S1). It is likely that the underlying genetic defect could account for the discrepancies in the experimental outcomes between the current study and the aforementioned published evidence. Han:SPRD rats are not a homologous model of human ADPKD having a missense mutation in Anks6 gene (Nagao et al., 2010), which causes Nephronophthisis (NPHP)‐like renal pathology in humans (Taskiran et al., 2014). The cystic disease in Han:SPRD rats has dominant inheritance and is characterized by a rapid development of cortical cysts with 75% of them originating from the proximal tubule (Schafer et al., 1994). In contrast, a homologous ARPKD model, PCK453 rat, develops cysts almost exclusively in the collecting duct system (Mason et al., 2010). Interestingly, no beneficial effects of high dietary citrate were found in pcy/pcy mice also having recessive inheritance of the disease (Tanner & Tanner, 2005). In fact, an increased kidney‐to‐bodyweight ratio was observed when potassium/citrate was given in drinking water (Tanner et al., 2000). This is consistent with the effects of high KB/C diet on kidney size shown in Figure 3a. Importantly, TRPV4 antagonist, GSK2193874, recapitulates the detrimental effect of high KB/C diet on kidney structure (i.e., augmented cystogenesis) suggesting an important role of the channel dysfunction in this case (Figures 3, 4, 5). Thus, the opposite effects of dietary citrate in Han:SPRD and PCK453 rats likely reflect the different function and localization of TRPV4 in proximal tubule and collecting duct. In the proximal tubule, TRPV4 is predominantly expressed on the basolateral side where it is not positioned to directly sense changes in tubular flow and regulate basal [Ca^2+^]_*i*_ levels, but rather serve as a pressure sensor of the filtration rate (Gualdani et al., 2020; Janas et al., 2016).

In summary, we demonstrate that TRPV4 function in cystic cells is an important determinant of growth of the collecting duct derived cysts in ARPKD rat model. Currently, we do not have direct experimental evidence showing that pharmacological or dietary (high KCl diet) stimulation of TRPV4 also attenuates the progression of clinically prevalent ADPKD, but it would likely be the case considering the collecting duct origin of the majority of cysts (Bergmann et al., 2018) and similar mechanisms of TRPV4 dysfunction in both PKD types (Tomilin et al., 2018). TRPV4 is expressed in different tissues and organs with the highest abundance in the kidney (Liedtke et al., 2000). This would provide at least a certain level of specificity upon systemic targeting TRPV4 activity with pharmacology. Previously, we provided a proof‐of‐principle evidence that long‐term administration of a low dose of TRPV4 agonist, GSK1016790A slowed renal cystogenesis in PCK453 rats with no apparent adverse effects (Zaika et al., 2013). Here, we boost this argument by demonstrating that physiologically relevant stimulation of TRPV4 activity with high KCl diet is an effective tool to counteract cyst formation. It should be noted that this maneuver stimulates channel expression/activity in the kidney, but not in other tissues endogenously expressing TRPV4. Thus, we posit that such strategy could be used alone or in combination with other drugs, such as tolvaptan, as an effective treatment of ARPKD and likely ADPKD in clinic.

## AUTHORS CONTRIBUTIONS

Conceptualization: Oleh Pochynyuk; investigation: Kyrylo Pyrshev, Anna Stavniichuk, Viktor N. Tomilin, Naghmeh Hassanzadeh Khayyat, Guohui Ren, Oleg Zaika, Mykola Mamenko, Oleh Pochynyuk; formal analysis: Kyrylo Pyrshev, Anna Stavniichuk, Viktor N. Tomilin, Naghmeh Hassanzadeh Khayyat, Guohui Ren, Mariya Kordysh, Oleg Zaika, Oleh Pochynyuk; funding acquisition: Kyrylo Pyrshev, Oleh Pochynyuk; writing original draft: Oleh Pochynyuk; editing: Kyrylo Pyrshev, Anna Stavniichuk, Viktor N. Tomilin, Naghmeh Hassanzadeh Khayyat, Guohui Ren, Mariya Kordysh, Oleg Zaika, Mykola Mamenko, Oleh Pochynyuk.

## CONFLICT OF INTEREST STATEMENT

The authors have no conflicts of interest to report.

## ETHICS STATEMENT

Animal use and welfare adhered to the NIH Guide for the Care and Use of Laboratory Animals following protocols reviewed and approved by the Animal Welfare Committee of the University of Texas Health Science Center at Houston.

## Supporting information


Figure S1–S5

Figure S1–S5
