# Supplementary figures and images for: TRPV4 functional status in cystic cells regulates cystogenesis in autosomal recessive polycystic kidney disease during variations in dietary potassium
Source: Physiol Rep. 2023 Mar 22;11(6):e15641. doi: 10.14814/phy2.15641 (PMC10031299; doi:10.14814/phy2.15641)

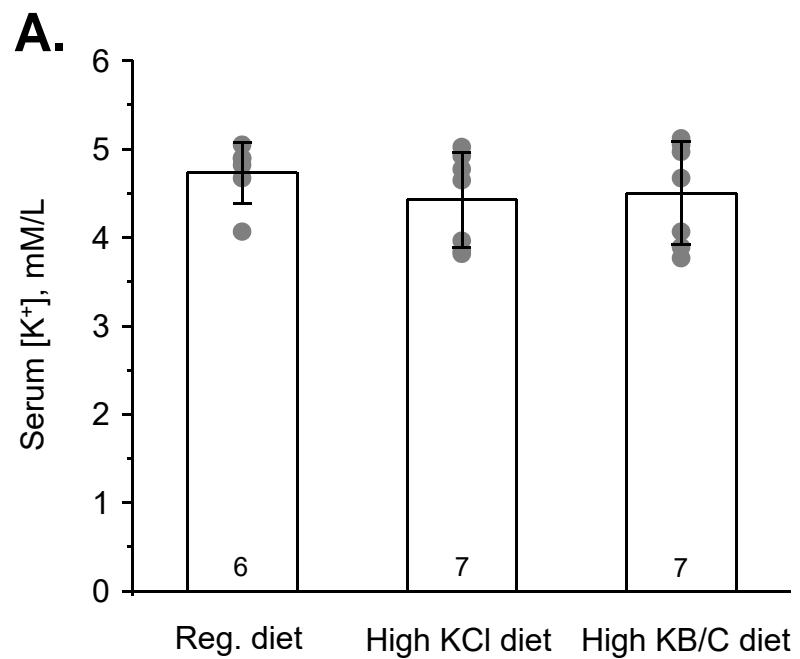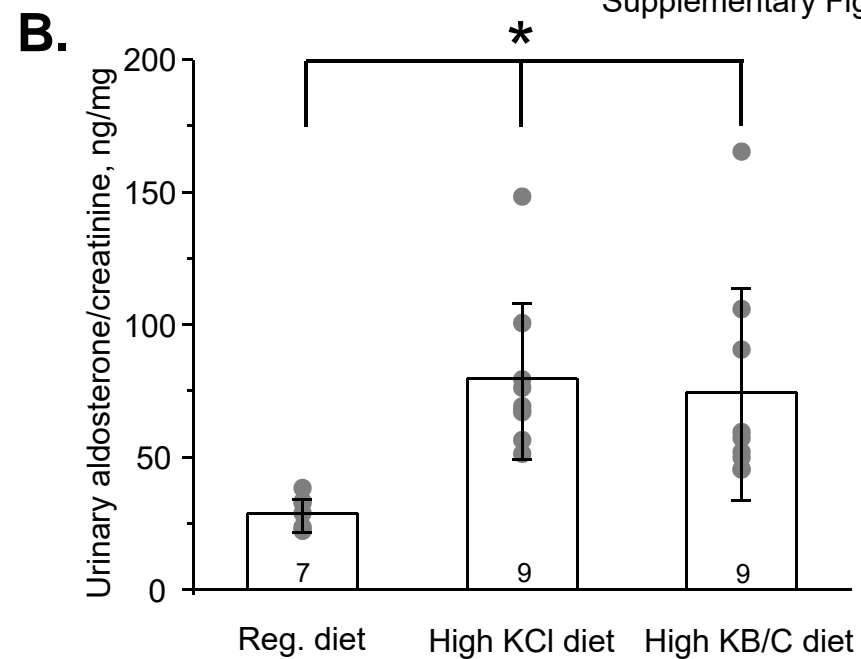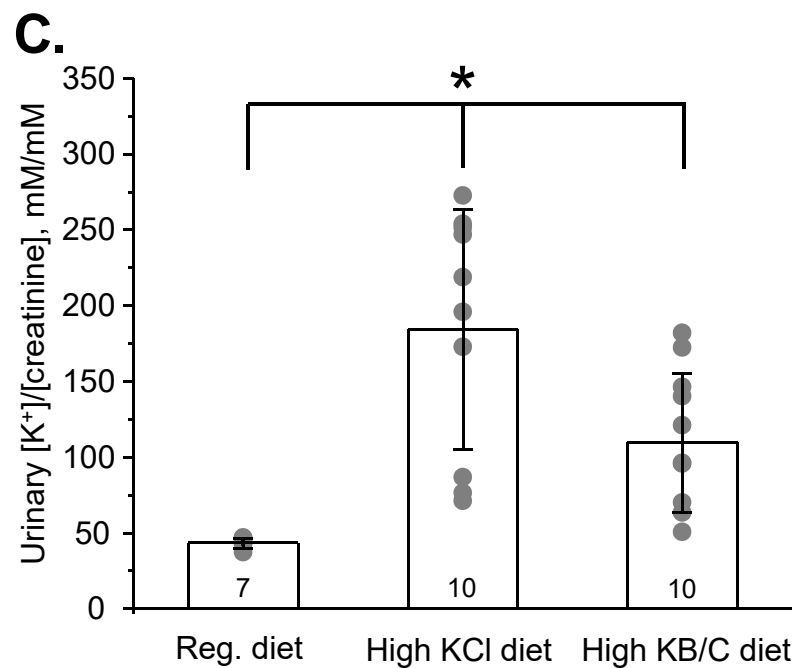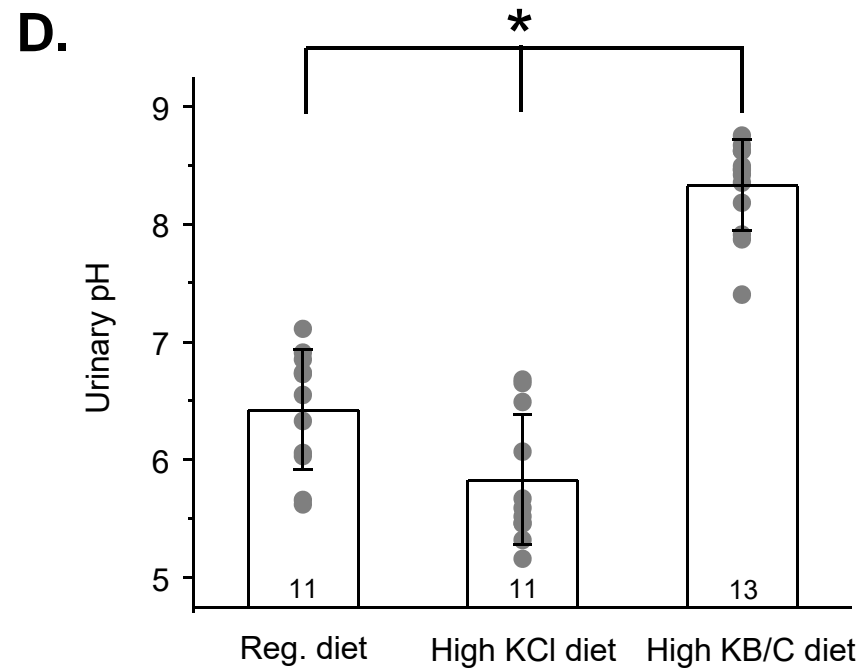

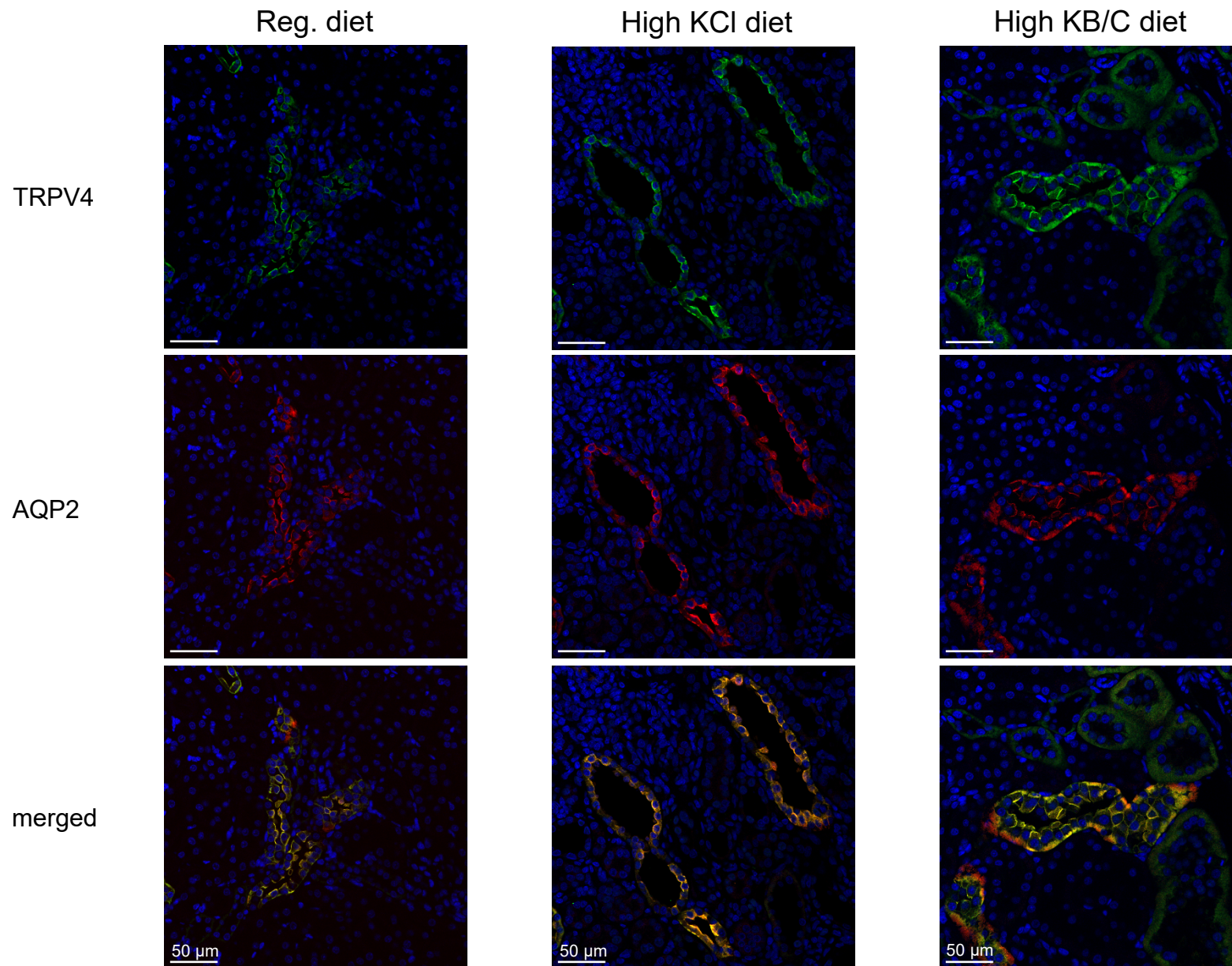

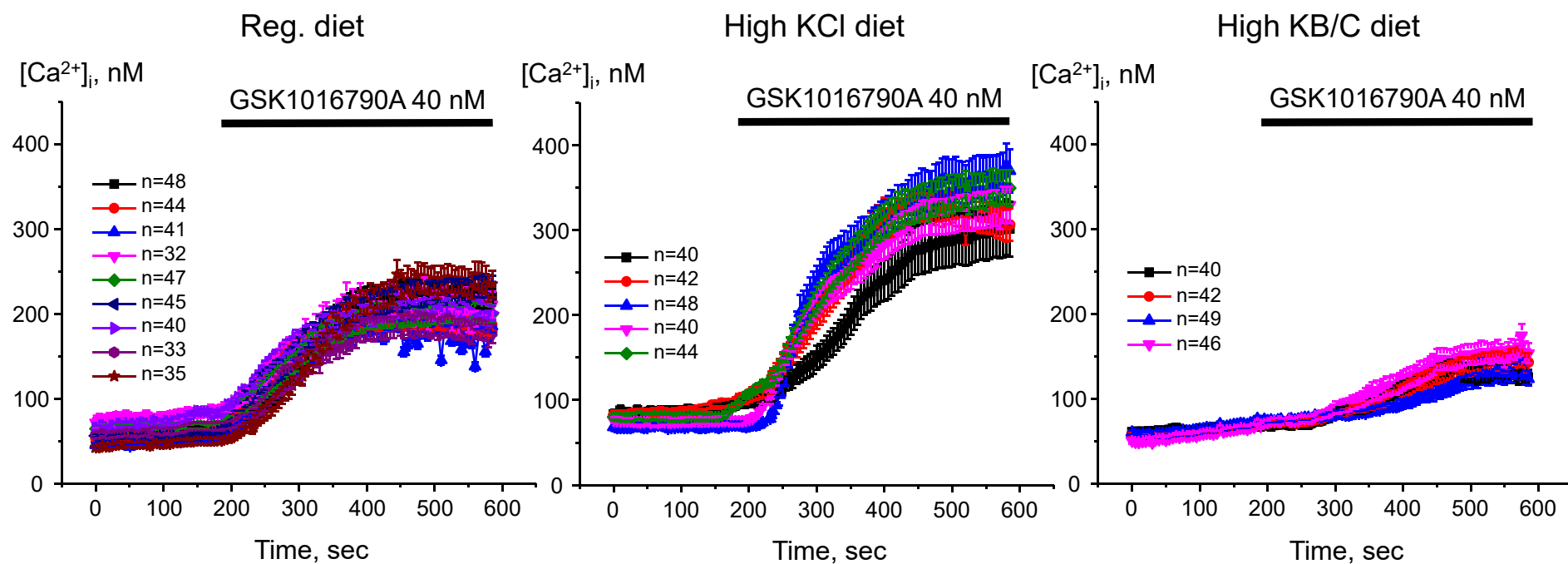

Supplementary Figure 4

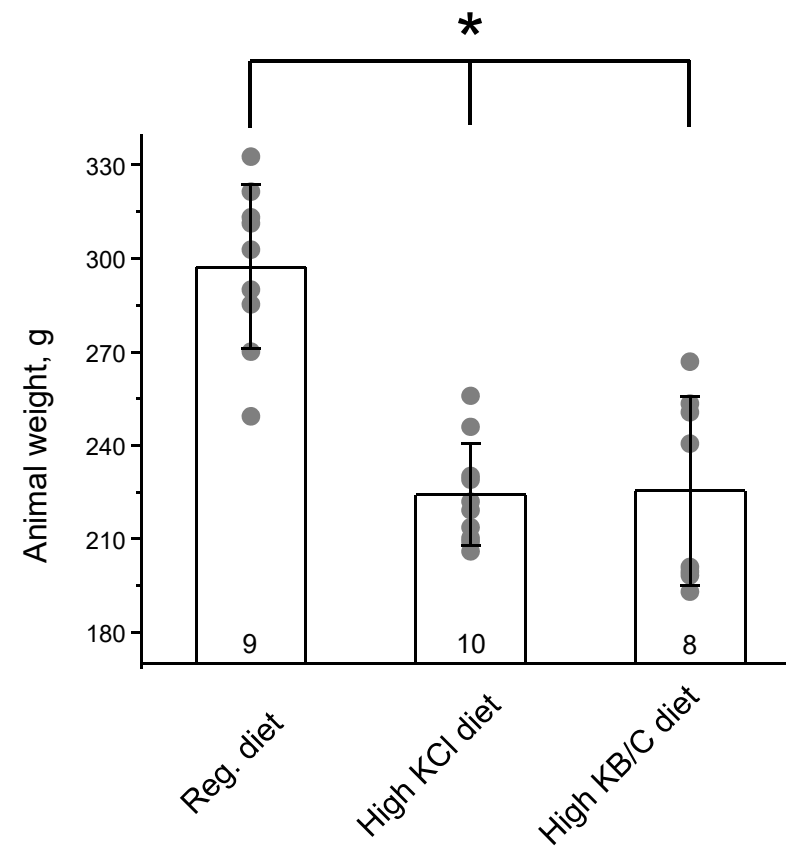

TRPV4

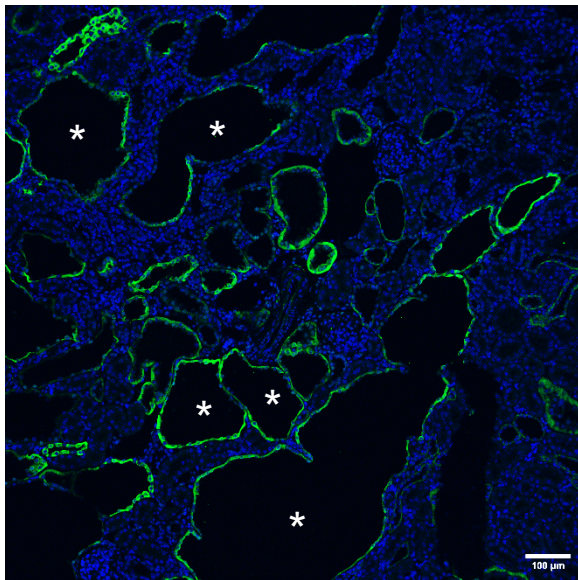

AQP2

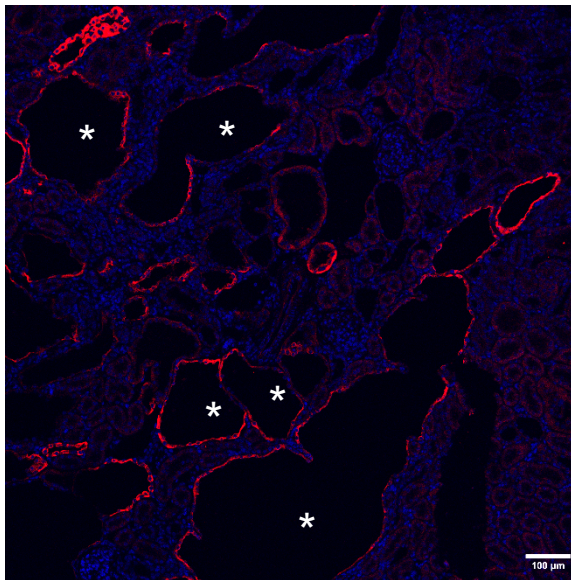

merged

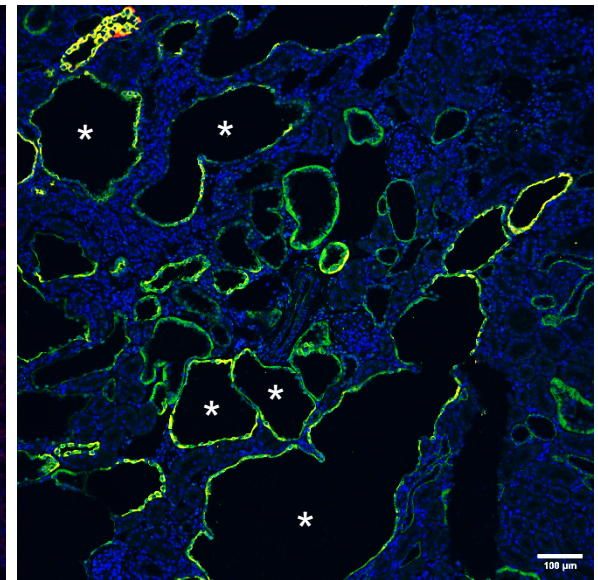

Supplement: Supplementary file 1 — Figure S1–S5 [file PHY2-11-e15641-s001.pdf]
