# Supplementary material for: TRPV4 functional status in cystic cells regulates cystogenesis in autosomal recessive polycystic kidney disease during variations in dietary potassium
Source: Physiol Rep. 2023 Mar 22;11(6):e15641. doi: 10.14814/phy2.15641 (PMC10031299; doi:10.14814/phy2.15641)
Supplement: Supplementary file 2 — Figure S1–S5 [file PHY2-11-e15641-s002.docx]

**SUPPLEMENTARY FIGURES**

**Figure S1. Characterization of systemic K^+^ balance in PCK453 rats fed high K^+^ diet.** Summary graphs comparing plasma K^+^ levels **(A)**, aldosterone to creatinine ratio in urine **(B)**, ratio of K^+^ to creatinine in urine **(C)**, and urinary pH **(D)** in PCK453 rats kept on regular (0.9%K^+^), high KCl (5%K^+^), and high KB/C (5%K^+^, bicarbonate : citrate as 4:1) diets for 1 month. Data are presented as mean ± SD. Numbers of each experimental groups (individual animals) are shown below. * - significant changes (P < 0.05, one-way ANOVA with post-hoc Tukey test) between experimental groups shown with brackets on the top.

**Figure S2. Regulation of TRPV4 localization in non-dilated renal tubules by dietary K^+^ intake.** Representative confocal micrographs showing TRPV4 (pseudocolor green), AQP2 (pseudocolor red), and the merged image in renal sections of PCK453 rats kept on regular (0.9%K^+^), high KCl (5%K^+^), and high KB/C (5%K^+^, bicarbonate : citrate as 4:1) diets for 1 month. Nuclear Dapi staining is shown with pseudocolor blue.

**Figure S3. TRPV4 stimulation induces reproducible [Ca^2+^]_i_ elevations in freshly isolated monolayer of cystic cells.** The averaged time time-courses of [Ca^2+^]_i_ changes upon application of 40 nM GSK1016790A (shown with the bar on top) in individual monolayers isolated from open cyst cavities from PCK453 rats kept on Regular (0.9%K^+^), high KCl (5%K^+^), and high KB/C (5%K^+^, bicarbonate : citrate as 4:1) diets for 1 month, as indicated. Different colors represent different monolayers with the number of analyzed cells is shown for each monolayer. The overall analysis of different cells for each condition is shown in Figure 2B.

**Figure S4. High KCl and high KB/C diets similarly decrease whole body weight in PCK453 rats.** Summary graphs comparing total bodyweight in PCK453 rats kept on regular (0.9%K^+^), high KCl (5%K^+^), and high KB/C (5%K^+^, bicarbonate : citrate as 4:1) diets for 1 month. Data are presented as mean ± SD. Numbers of each experimental groups (individual animals) are shown below. * - significant changes (P < 0.05, one-way ANOVA with post-hoc Tukey test) between experimental groups shown with brackets on the top.

**Figure S5. Cortical dilations have collecting duct origin in PCK453 rats fed high KB/C diet.** Representative confocal micrographs showing TRPV4 (pseudocolor green), AQP2 (pseudocolor red), and the merged image in renal cortex of a PCK453 rat kept on high KB/C (5%K^+^, bicarbonate : citrate as 4:1) diets for 1 month. Nuclear Dapi staining is shown with pseudocolor blue.
